# Oral Health in Japan: State-of-the-Art and Perspectives

**DOI:** 10.3390/ijerph19148232

**Published:** 2022-07-06

**Authors:** Masanobu Abe, Akihisa Mitani, Atsushi Yao, Liang Zong, Chun-Dong Zhang, Kazuto Hoshi, Shintaro Yanagimoto

**Affiliations:** 1 Division for Health Service Promotion, The University of Tokyo, Tokyo 113-8654, Japan; mitania-int@h.u-tokyo.ac.jp (A.M.); yaoa-int@h.u-tokyo.ac.jp (A.Y.); yanagimoto@hc.u-tokyo.ac.jp (S.Y.); 2 Department of Oral & Maxillofacial Surgery, The University of Tokyo Hospital, 7-3-1 Hongo, Bunkyo-ku, Tokyo 113-8655, Japan; hoshi-ora@h.u-tokyo.ac.jp; 3 Division of Epigenomics, National Cancer Center Research Institute, Tokyo 104-0045, Japan; zl20014111@163.com (L.Z.); zhangchundong2007@126.com (C.-D.Z.); 4 Department of Gastrointestinal Surgery, Graduate School of Medicine, The University of Tokyo, Tokyo 113-8654, Japan

In the near future, Japan is entering a super-aging society that will be called the age of 100 years of life [1]. As of 2019, the average life expectancy of the Japanese is 81.5 years for men, ranking second in the world, and 86.9 years for women. This ranks first in the world as a whole, making Japan the longest-living country in the world [2]. Although the number of deaths among the elderly has been decreasing, the birth rate has been declining: the working-age population supporting one elderly person aged 65 or older was 2.3 persons per elderly person in 2015 and is expected to be 1.3 persons per elderly person in 2065. An aging society with a declining birthrate is likely to cause a variety of problems. The most serious problem is the collapse of the balance between benefits and burdens related to social security, especially the balance between medical and long-term care costs [3]. In order to minimize the increased burden on the working-age population and maintain the quality of life of the elderly, it is necessary for the entire nation to work toward extending the healthy life expectancy of the population. Recently, it has been shown that people who have more teeth have a longer healthy life expectancy and require less nursing care [1]. Maintaining healthy teeth and periodontal tissues and eating with one’s own teeth even in old age is not only good for one’s personal health, but also for the well-being of Japanese society as a whole.

Oral diseases are of critical public health importance in terms of their high prevalence. The disease with the highest prevalence in the world is untreated permanent tooth caries (35%). Severe periodontitis is also reported to have the sixth highest prevalence in the world (11%) [4]. In Japan, caries has been the biggest target of oral diseases, and its treatment and prevention have been long-standing issues [5]. Since the 1980s, however, caries has been greatly reduced due to the spread of fluoride and educational activities by the Japanese Dental Association, the Ministry of Health and Welfare (now the Ministry of Health, Labor, and Welfare), the 8020 Promotion Foundation, and other dental professionals. The incidence of dental caries (decayed-filled teeth) among adolescents (15–19 years old), for whom caries prevention is particularly important, decreased significantly from 94.9% in 1993 to 47.1% in 2016 [5]. Although dental caries used to be the main cause of tooth loss [6,7], periodontal disease is currently the leading cause (40.4% in men and 34.9% in women), surpassing caries (30.2% in men and 29.0% in women) [8]. Although caries control must continue, we need to turn our attention to the next target “periodontal disease” in anticipation of a super-aging society. One of the biggest differences between dental caries and periodontal disease is the presence or absence of pain. Whereas caries causes severe pain as it progresses, periodontal disease usually causes symptoms, such as discomfort and gingival bleeding, but does not cause severe pain [9]. By the time the disease is noticed, the periodontal tissue has been destroyed and the teeth become loose and fall out. Periodontal disease is a silent disease of the oral cavity.

The symptoms of periodontal disease usually become apparent in people in their 40s or later in Japan, and, therefore, the target group for treatment is middle-aged and older adults. However, young people also often develop gingivitis, an early stage of periodontal disease; 30.6% of 15- to 19-year-olds complained of gingival bleeding in the Ministry of Health, Labor, and Welfare’s survey of dental diseases [5]. In a survey of University freshmen, 36.5% of 17- to 19-year-olds complained of gingival bleeding [10]. This means that approximately one out of three people in their late teens already have early signs of periodontal disease. It has also been shown that periodontal pockets rapidly become deeper after the age of 20. The percentage of those with periodontal pockets 4 mm or deeper was 6.1% for those aged 15–19 years, while 25.7% for those aged 20–24 years, showing a sharp increase after the age of 20 [5]. This result indicates that periodontal disease already begins in adolescence, suggesting the need for periodontal disease control not only in middle-aged and older adults but also in adolescents [11].

More importantly, it has become clear that periodontal disease not only causes looseness of tooth, but is also closely associated with systemic health [12]. The association of periodontal disease with various diseases has been reported, including cardiovascular diseases [12,13,14,15,16,17,18,19,20,21], respiratory diseases [22,23,24,25], rheumatoid arthritis [26,27], diabetes mellitus [28,29], osteoporosis [30], metabolic syndrome [31], Alzheimer’s disease [32,33], preterm birth, and fetal growth restriction [34,35]. The association between periodontal disease and systemic diseases usually becomes apparent in middle-aged and older adults after the age of 40, but recently it has been shown that gingival bleeding, an early symptom of periodontal disease, is closely associated with systemic diseases, such as asthma and otitis, in adolescents [10]. In addition to periodontal disease and dental caries, the association between malocclusion and systemic diseases has also been suggested in recent years [36,37,38]. Therefore, it is no longer easy to separate oral diseases from systemic health. Close and deepened cooperation between medicine and dentistry is required.

Oral hygiene status and self-care (brushing) are closely related to periodontal disease [39,40]. When the gingival margin is not well cleaned, plaque stagnates and bacteria grow in it, causing inflammation, and bleeding begins from the gingiva [41]. Our recent findings indicate that the risk of gingival bleeding in adolescents (17–19 years of age) is higher the less frequently they brush their teeth and the shorter the brushing time. The risk of gingival bleeding was 1.5 times higher in those who brushed their teeth twice a day compared with those who brushed three or more times a day, and 2.4 times higher in those who brushed once a day or less compared with those who brushed three or more times a day. The risk of gingival bleeding was 1.3 times higher in those who brushed their teeth 2–3 min per each time compared with those who brushed for 4 min or more, and 1.6 times higher in those who brushed for 1 min or less per each time compared with those who brushed for 4 min or more [42]. This result suggests that frequent and time-consuming brushing can prevent gingival bleeding. In the early stages of periodontal disease, “gingivitis,” it is possible to restore normal periodontal tissue through improved oral hygiene. To this end, it is necessary to actively educate the public, especially the younger generation, to practice and improve self-care for the prevention of gingivitis [43].

The key to combating periodontal disease is prevention and early detection. Regular dental health checkups and health guidance from adolescence, rather than from middle age, can control gingivitis in its early stages and reduce its progression to periodontitis. Furthermore, it can lead to the prevention of the onset and severity of systemic diseases that have been suggested to be associated with periodontal disease. A large data analysis has reported that having periodontal checkups and treatment not only maintains periodontal health but also reduces the incidence of ischemic stroke [44]. Legally mandated dental checkups include: 1-year, 6-month and 3-year-old checkups under the Maternal and Child Health Act, school dental checkups for school children under the School Health and Safety Act, periodontal disease checkups at ages 40, 50, 60 and 70 under the Health Promotion Act, and special dental checkups for harmful work defined by the Industrial Safety and Health Act, such as acid erosion, etc. In addition, dental checkups for the elderly are provided based on the Act on Securing Medical Care for the Elderly. However, the working-age population from high school graduation (age 18) to age 40, have no opportunities to receive dental checkups except for special checkups for limited target occupations. Considering that periodontal disease begins in adolescence and becomes apparent in one’s 40s, it is also important to fill the gap in the dental checkup from high school graduation to age 40. In Japan, where the population is aging rapidly, the main goal is to increase the healthy life expectancy to the average life expectancy. To achieve this goal, it is essential to establish a seamless oral health management system that is appropriate for each stage of life.
